# School Feeding and Girls’ Enrollment: The Effects of Alternative Implementation Modalities in Low-Income Settings in Sub-Saharan Africa

**DOI:** 10.3389/fpubh.2015.00076

**Published:** 2015-05-20

**Authors:** Aulo Gelli

**Affiliations:** ^1^Poverty, Health and Nutrition Division, International Food Policy Research Institute, Washington, DC, USA

**Keywords:** evaluation, school feeding, enrollment

## Abstract

**Background:**

School feeding interventions are implemented in nearly every country in the world, with the potential to support the education, health and nutrition of school children. In terms of impact on school participation, there is little evidence to show that different school feeding modalities have different effect sizes.

**Objective:**

To examine the influence of different school feeding modalities on primary school enrollment, particularly for girls, in 32 countries across sub-Saharan Africa.

**Methods:**

An observational study involving a meta-analysis of published data was developed to examine program effect. Schools were divided according to the type and length of the program: those with existing programs, those that had had school feeding for less than 1 year, and a counterfactual including schools without a program but that were going to initiate school feeding within the survey year. The intervention consisted of two different types of school feeding: onsite meals alone or onsite meals plus take-home rations. Changes in enrollment, both total and disaggregated by grade and gender, over a 1-year period, were used to assess effects of school feeding. To control for pre-program characteristics in the beneficiary population, data on covariates were also examined before the school feeding intervention began and after one year of implementation. Using this design a comparison of enrollment levels was made between the types of treatment schools and controls schools during the period school feeding was first introduced. Standard multiple regression models were used to analyze program effect.

**Results:**

School feeding programs were found to have statistically significant increases in enrollment, with effect size of about 10%. The changes on enrollment varied by modality of school feeding provision and by gender, with onsite meals appearing to have stronger effects in the first year of treatment in the lower primary grades, and onsite combined with take-home rations also being effective post-year 1, particularly for girls that were receiving the extra take-home rations.

**Conclusion:**

School feeding programs had a positive impact on school enrollment. The operational nature of the survey data used in the meta-analysis, however, limits the robustness of the design and validity of the findings. Nevertheless, this analysis is the first to study possible links between enrollment and length of program duration using multivariable models, examining whether programs reach a saturation point or steady state beyond which school feeding may in fact have no further benefits on school enrollment. Further research is required to examine this issue in more detail.

## Introduction

The last decade has seen remarkable improvements in terms of access to primary education across many low- and middle-income countries (1). Yet challenges remain and 58 million children of primary school-age are still not in school (2). Poor nutrition and health among schoolchildren contributes to the inefficiency of the educational system (3). Children with diminished cognitive abilities perform less well and are more likely to repeat grades and to drop out of school; they also enroll in school at a later age and finish fewer years of schooling (4). Short-term hunger in children may result in difficulty in concentrating and performing complex tasks (5).

The scale-up of school feeding programs has been a key education sector response to the recent economic crises (6). Analysis of program scale suggests that every country in the world is seeking to feed its school children, though coverage is weakest in low-income countries (7). The evidence base on the educational benefits of school feeding has been summarized in a number of recent reviews (7–9): School feeding programs can help to get children into school and help to keep them there, increasing enrollment and reducing absenteeism; and once the children are in school, the programs can contribute to their learning, through avoiding hunger and enhancing cognitive abilities. In practice however, school feeding programs are complex interventions with many possible configurations, involving a broad range of activities by different stakeholders at different levels (10).

There is little evidence to show that different school feeding modalities have different effect sizes on school participation, whether we consider enrollment, attendance, or drop-out (8). Two recent randomized control trials (RCTs) examine the differential impact of school feeding on child development outcomes. In northern Burkina Faso, a RCT assessed the impacts of two alternative school feeding interventions, onsite meal and take-home rations, on enrollment, academic performance, cognitive development and pre-school children nutritional status (11). Both onsite meals and take-home rations were found to increase enrollment by 6%. Similarly, a RCT set in internally displaced people camps in Northern Uganda found that both school meals and take-home rations had a positive impact on school participation, including enrollment for children not enrolled prior the introduction of school feeding, and on morning and afternoon attendance (12).

Despite its popularity as a program, there is very little evidence on the costs of school feeding. The available evidence suggests that the different modalities have very different costs (13). This raises important questions in terms of cost-effectiveness and sustainability. Policy makers and implementers can therefore benefit from a careful assessment of the trade-offs associated with different program designs.

This paper is aimed at examining the influence of different school feeding modalities, namely onsite feeding or onsite feeding combined with take-home rations, on primary school enrollment, particularly for girls, in 32 countries across sub-Saharan Africa. This study involves a meta-analysis of perhaps the largest school-level dataset collected on school feeding programs across sub-Saharan Africa, covering 32 countries. It builds on a previous study undertaken in 2007 that focused on describing average changes in schools with and without school feeding (14). In this paper, the original dataset is developed to include a range of school-level variables and an analysis of program effect is undertaken using a new estimation strategy.

## Methodology

The starting point for the data used in this analysis was a database used in a previously published study (14). The original dataset involved an aggregation of school-level surveys collected from a centralized database based at the World food Program (WFP) head-quarters in Rome. The study population consisted of all WFP-targeted primary schools, which are generally located in areas vulnerable to food insecurity and poor access to education.

### Data

In each of the surveys included in this study, the sample of schools was selected either by simple random sampling or by using random sampling with probabilities proportional to school size. In some countries with small programs a school census was undertaken. Sample frames were stratified by program duration: schools with recently introduced school feeding programs and schools with school feeding programs that had been operating for 1 year or more. Details of survey implementation are published elsewhere (14). A number of different questionnaires were used throughout the survey period that spanned 2002–2005, though core sections covering enrollment information were retained in all versions. In order to examine short-term trends, each survey collected enrollment data over 3 years. Surveys involved semi-structured, school-level questionnaires that included interviews with school heads, teachers, parents, and pupils, covering educational indicators, particularly enrollment and attendance. Data on school infrastructure, classrooms, teaching, and other school quality-related indicators were also collected to monitor the program context.

## Data Extraction

Data from the different surveys were exported from a centralized WFP database and merged. As the questionnaires used in the different countries varied throughout the survey period, the first step in the analysis involved a harmonization of the different data to match variable definitions. A number of additional school- and country-level variables were then added to the dataset. School-level variables included number of classrooms and number of teachers. Country-level variables were added to examine cross-country variability, including indicators that are linked to the main aims of school feeding programs, covering measures of poverty, undernutrition, and education. Poverty was captured by per capita Gross Domestic Product (GDP) (Source: World Bank), undernutrition was captured through the prevalence of undernourishment (Source: FAO), and levels of education were measured through the primary school net enrollment ratio (NER) (Source: UNESCO). In practice, due to inconsistencies in the indicator definitions it was not possible to aggregate all the school-level data collected throughout the 5 years. As a result, this study focuses on the analysis of trends in absolute enrollment observed throughout the survey years; full results for each of the different surveys have been published elsewhere (15). Table 1 shows details of the surveys included in this analysis.

**Table 1:** **Survey details, in 32 countries in sub-Saharan Africa**.

	Assisted schools	Beneficiaries in 2004^a^	Schools in sample	Total pupils in sample	Survey start	Survey end
Angola	718	37,189	224	56,760	10/10/2003	19/05/2004
Burkina Faso	210	40,384	51	4,933	02/06/2003	11/07/2003
Burundi	73	24,669	66	35,446	04/06/2003	05/06/2003
Cameroon	386	134,648	146	33,466	08/02/2004	17/02/2005
Cape Verde	417	107,477	59	12,150	02/10/2003	09/10/2003
C. A. R.	143	104,023	83	17,904	17/01/2004	09/02/2004
Chad	350	73,539	104	14,279	18/11/2002	19/02/2003
Congo Republic	72	16,608	72	14,567	13/10/2003	13/11/2003
D. R. Congo	170	95,521	170	109,834	02/10/2003	22/11/2003
Djibouti	50	10,963	48	10,561	01/05/2003	17/12/2003
Eritrea	212	126,848	122	46,308	07/10/2003	27/11/2003
Ethiopia	626	736,305	188	122,635	04/11/2002	19/12/2002
Ghana	323	34,368	60	9,461	26/01/2003	27/02/2003
Guinea	435	175,365	185	24,223	04/10/2003	22/10/2003
Guinea Bissau	487	124,537	124	11,577	07/06/2003	02/07/2003
Kenya	4,388	1,491,433	330	100,070	03/11/2003	28/11/2003
Lesotho	1281	176,393	206	60,008	02/03/2003	30/04/2003
Liberia	602	387,630	211	41,122	01/03/2004	03/08/2004
Malawi	201	219,017	122	102,081	11/02/2003	10/04/2003
Mali	330	91,318	123	28,992	31/03/2003	22/06/2003
Mauritania	1,122	78,200	176	20,548	07/12/2003	28/12/2003
Mozambique	164	275,474	127	91,872	10/03/2003	11/04/2003
Niger	242	38,777	110	11,929	05/03/2003	15/05/2003
Rwanda	338	179,165	124	88,802	21/05/2003	16/06/2003
S. Tome & Principe	74	28,280	42	10,193	27/10/2003	11/12/2003
Sénégal	998	250,680	277	32,457	16/02/2004	24/02/2004
Sierra Leone	729	277,386	256	78,526	15/06/2003	19/11/2003
Somalia	31	7,220	31	5,180	09/03/2003	18/03/2003
Tanzania	215	170,843	60	23,916	10/12/2002	22/01/2003
The Gambia	340	132,902	84	37,082	21/04/2004	04/05/2004
Uganda	432	470,956	199	95,290	14/02/2003	26/03/2003
Zambia	180	92,409	104	45,589	16/10/2003	31/10/2003

*^a^The number of beneficiaries was taken from the WFP 2005 Standardized Project Reports (SPRs) is in some cases be greater than the number of students in the sample. This is due to the fact that the survey year of reference does not correspond with that of the SPRs*.

*Source: Gelli et al. (14)*.

The sample covered a total of 4,175 schools, including 903 schools without a school feeding program, 593 schools that had just started school feeding, and 2,680 schools that had received school feeding for over a year (14). The data did not differentiate schools with only take-home rations from those providing take-home rations combined with onsite feeding; as a result, an assumption was made in this analysis that all programs with take-home rations also offered onsite feeding. This assumption is justified by the fact that WFP programs in sub-Saharan Africa that provide take-home rations do so in combination with regular onsite feeding (13). It is important to note that in combined programs take-home rations are provided to girls only, whilst meals are provided to all children regardless of gender.

### Estimation strategy

The ideal strategy for estimating the impact of school feeding programs would involve randomly assigning similarly eligible children and communities to the intervention and control groups (16). However, in this case the random assignment to WFP school feeding programs, particularly in programs operating in food-insecure areas, has proven difficult to implement for logistical, ethical, and political reasons (15). This is an observational study, where a quasi-experimental design was developed as a next best possible option to examine program effect (17). In quasi-experimental designs, beneficiaries are compared to nonrandomly assigned controls that do not receive an intervention. In this analysis schools were divided according to the type and length of the program: those with existing programs, those that had had school feeding for less than 1 year, and a counterfactual including schools without a program but that were going to initiate school feeding within the survey year.

The intervention consisted of two different types of school feeding: either onsite meals alone or onsite meals combined with take-home rations. Schools were selected by WFP for school feeding programs using very similar criteria across countries, primarily on the basis of geographical targeting based on food security and education indicators. Changes in absolute enrollment, both total and disaggregated by grade and gender, over a 1-year period, were used to assess possible effects of school feeding. To control for pre-program characteristics in the beneficiary population, data on covariates were also examined before the school feeding intervention began and after 1 year of implementation. Using this design a comparison of enrollment levels was made between the two types of treatment schools and controls schools during the period school feeding was first introduced. Comparisons were also made between the schools that had the two different types of treatments at baseline. Standard multiple regression models were used to analyze program effect, using the following equation:

$$\begin{array}{lll} \frac{y_{\text{t2}}-y_{\text{t1}}}{y_{\text{t1}}}=\propto_{0}+\sum_{\text{i}=1}^{4}\propto_{\text{i}}P_{\text{i}}+\propto_{5}g+\sum_{\text{i}=2}^{5}\propto_{\text{i}+4}\text{g}{\text{r}}_{\text{i}}+\sum_{\text{i}=1}^{4}\propto_{\text{i}+9}P_{\text{i}}*g+\propto_{14}P_{1}*\text{g}{\text{r}}_{\text{2}}+\propto_{15}P_{1}\; & & \;\\ \;\;\text{∗ g}{\text{r}}_{\text{3}}+...+\propto_{29}P_{4}\text{g}{\text{r}}_{\text{5}}+\propto_{30}\text{ pcr}+\propto_{31}\text{ ptr}+\propto_{32}{\;e}_{\text{t}1}+\propto_{33}\text{GDPc}\; & & \;\\ \;\;+\propto_{34}\text{ NER}+\propto_{35}\text{ UND}+\varepsilon \; & & \; \end{array}$$

where *y*_t_ is the absolute enrollment at time t; *P*_i_ a is a dummy variable for program type (*P*_1_ = 1 for onsite meal first year, *P*_2_ = 1 for onsite meal plus take home ration programs first year, *P*_3_ = 1 for onsite meal after first year, *P*_4_ = 1 for onsite meal plus take home ration programs first year, else *P*_i_ = 0); *g* is a dummy variable for gender (*g* = 1 for girls, 0 for boys) gr_i_ is a dummy variable for primary school grade (gr_2_ = 1 for grade 2, gr_3_ = 1 for grade 3, gr_4_ = 1 for grade 4, gr_5_ = 1 for grade 5, else gr_i_ = 1); pcr is the pupil-to-classroom ratio; ptr is the pupil-to-teacher ratio; and *e*_t1_ is the enrollment level at baseline. GCPc is the country-level GDP per capita, NER the country-level primary net enrollment ratio, and UND is the country-level prevalence of undernourishment.

### Validity of estimation strategy

There are a number of important concerns regarding the validity of the estimation strategy in this analysis. The extent to which this quasi-experimental evaluation will result in unbiased estimates of program impact will largely depend on whether the allocation of treatment to the different groups was independent of characteristics that are correlated with the outcome measurements, and also on observable (and unobservable) differences between the control and intervention groups pre-intervention. Considering the issue on internal validity and selection bias, schools assisted by WFP school feeding programs are generally targeted using similar criteria based on food insecurity and vulnerability analysis and mapping, as well as an analysis of the educational context in each country. As the intervention was not undertaken for the purpose of research and the assignment to the different treatment arms was not randomized, this analysis design can be characterized as a natural experiment (18). As a result, it is important to identify confounders and minimize bias when estimating program effect. With regards to the comparability of the schools, Table 2 summarizes the available school-level pre-intervention characteristics. Only a small number of covariates at “baseline” (t1 in the estimation strategy) could be extracted from the survey data, as most of the questionnaires collected data for the survey year (or t2 in the estimation strategy). The school-level covariates included the pupil-to-classroom ratio (the number of children enrolled in a school, as listed in the school register at the beginning of the school year, divided by the number of classrooms in the school) and the pupil-to-teacher ratio (the number of children enrolled in a school, as listed in the school register at the beginning of the school year, divided by the number of teachers registered to work in the school). A broader set of school-level indicators were available at “follow-up” (t2). A comparison of the observable school characteristics of the two groups at (t1) shows a statistically significant difference in pupil-to-classroom ratios (Table 2).

**Table 2:** **Public primary school-level characteristics at study “baseline” (t1), sub-Saharan Africa, 2002–2005**.

School characteristics	Control			School feeding			Difference		
	Mean	[95% C. I.]		Mean	[95% C. I.]		Coef.	SE	*P*
Total enrollment	613	539	687	602	539	666	10	50	0.84
Girls enrollment	299	263	336	278	248	309	21	24	0.386
Boys enrollment	313	275	352	324	290	359	−10	26	0.681
Pupil-to-teacher ratio	59	57	62	58	55	62	1	2	0.556
Pupil-to-classroom ratio	67	62	72	84	77	90	−17	4	<0.001

Similarly, a comparison of school-level characteristics between schools with 1 year of either onsite meals or a combination of onsite and take-home rations shows a number of statistically significant differences that limit the internal validity of the analysis (Table 3). In this case, differences in school size, or school enrollment, are significantly different across the two groups.

**Table 3:** **Public primary school-level characteristics at study “baseline” for schools with existing school feeding programs, sub-Saharan Africa, 2002-2005**.

	Onsite meals more than 1 year			Onsite and THRs more than 1 year			Difference		
	Mean	[95% C. I.]		Mean	[95% C. I.]		Coef.	SE	*P* > *t*
Total enrollment	945	819	1071	666	597	734	279	73	<0.001
Girls enrollment	426	365	487	304	268	341	122	36	0.001
Boys enrollment	519	452	585	361	325	397	157	39	<0.001
Pupil-to-teacher ratio	63	61	66	71	67	75	−7	2	0.002
Pupil-to-classroom ratio	106	99	113	97	80	115	8	10	0.368

## Results

Due to a combination of different versions of questionnaires and survey years, only a very small number of school-level covariates were available alongside the outcome variables, including pupil-to-classroom and pupil-to-teacher ratios. The regressions were run using estimation weights, equal to the inverse of inclusion probabilities, using the “svy: reg” command in STATA. The results of the regressions model with enrollment as the dependent variable and the available school-level and country-level variables are shown in Table 4.

**Table 4:** **Effects of school feeding and other school-level variables on changes in school enrollment in public primary schools, sub-Saharan Africa, 2002–2005**.

Variable	Coef.	SD	*t*	*P*	[95% Conf. Interval]	
School feeding	0.0961	0.0212	4.54	<0.001	0.0546	0.1376
Baseline enrollment	−0.0002	0.0000	−11.22	<0.001	−0.0002	−0.0002
Pupil-to-teacher ratio	−0.0022	0.0005	−4.59	<0.001	−0.0031	−0.0012
Pupil-to-classroom ratio	−0.0004	0.0001	−3.02	0.003	−0.0006	−0.0001
Undernourishment	−0.0042	0.0013	−3.14	0.002	−0.0068	−0.0016
Net enrollment ratio	0.0060	0.0008	7.45	<0.001	0.0044	0.0076
GDP per capita	−0.0006	0.0001	−7	<0.001	−0.0007	−0.0004
Constant	0.6384	0.1098	5.81	<0.001	0.4231	0.8537
R-squared	0.0335					

School feeding programs were found to have statistically significant increases in enrollment, with effect size of about 10%. The coefficient for the enrollment levels at baseline showed a small, statistically significant association such that larger proportionate changes in enrollment are found in schools with lower baseline levels, a finding that is consistent with program experience. The negative coefficients on pupil-to-classroom and pupil-to-teacher ratios also highlight this effect. In terms of country-level variables, the prevalence of undernourishment and GDP per capita showed consistent and statistically significant negative effects on enrollment. The coefficients for primary net enrollment ratio were consistently positive and statistically significant, suggesting that changes in enrollment were higher in countries that were performing better in terms of schooling access. The regression model generally explained only just over 3% of the variability in the data, which is not surprising considering that the determinants of schooling tend to be driven by individual and household-level characteristics not covered in the models. This finding highlights the inadequacy of school feeding impact evaluations limited to school level data collection.

In order to examine the effects of school feeding in more detail, regressions were also run for enrollment controlling for type and duration of program (see Table 5). Enrollment levels in schools with onsite meal programs during the first year of treatment showed statistically significant increases of over 15%. Combined programs were found to increase enrollment after the first year of treatment, by about 8%.

**Table 5:** **Effects of school feeding and other school-level variables on changes in school enrollment in public primary schools, controlling for program type, sub-Saharan Africa, 2002–2005**.

Variable	Coef.	SE	*t*	*P* > *t*	[95% Conf. Interval]	
Onsite, 1st year	0.1537	0.0302	5.10	<0.001	0.0946	0.2128
Combined, 1st year	0.1083	0.0635	1.70	0.088	−0.0162	0.2329
Onsite, 2nd year	0.0291	0.0208	1.40	0.162	−0.0117	0.0698
Combined, 2nd year	0.0770	0.0245	3.14	0.002	0.0290	0.1251
Baseline enrollment	−0.0002	0.0000	−10.38	<0.001	−0.0002	−0.0001
Pupil-to-teacher ratio	−0.0023	0.0004	−5.34	<0.001	−0.0032	−0.0015
Pupil-to-classroom ratio	−0.0003	0.0001	−2.71	0.007	−0.0006	−0.0001
Undernourishment	−0.0033	0.0013	−2.47	0.014	−0.0058	−0.0007
Net enrollment ratio	0.0053	0.0008	6.84	<0.001	0.0037	0.0068
GDP per capita	−0.0006	0.0001	−6.84	<0.001	−0.0007	−0.0004
Constant	0.6777	0.1132	5.99	<0.001	0.4559	0.8996
R-squared	0.0369					

Regressions were also run to control for gender and school grade (see Table 6). The increases in enrollment in schools in the first year of onsite meals were found to vary mainly with primary school grade and not by gender. On the other hand, the effect of combined programs after the first year of treatment was driven by an increase in girls’ enrollment, which was found to be about 12% greater than the change in boys’ enrollment, with no statistically significant interactions across grades. The *F*-tests for the type of school feeding and gender interactions (*F* = 2.89, Prob. >*F* = 0.0209) and the type of school feeding and grade interactions (*F* = 2.96, Prob. >*F* = 0.0001) reject the hypotheses of zero coefficients at 5% significance level.

**Table 6:** **Effects of school feeding and other school-level variables on changes in school enrollment in public primary schools, controlling for program type, gender and grade, sub-Saharan Africa, 2002–2005**.

Variable	Coef.	SE	*t*	*P*	[95% Conf. Interval]	
Onsite, 1st year (sfy1)	0.4082	0.0935	4.36	<0.001	0.2249	0.5915
Combined, 1st year (thry1)	0.2003	0.3398	0.59	0.556	−0.4659	0.8664
Onsite, 2nd year (sfy2)	0.0634	0.0399	1.59	0.112	−0.0148	0.1415
Combined, 2nd year (thry2)	−0.0046	0.0421	−0.11	0.912	−0.0871	0.0778
Baseline enrollment	−0.0002	0.0000	−10.64	<0.001	−0.0002	−0.0001
Pupil-to-teacher ratio	−0.0023	0.0004	−5.37	<0.001	−0.0032	−0.0015
Pupil-to-classroom ratio	−0.0003	0.0001	−2.81	0.005	−0.0006	−0.0001
Gender	0.0337	0.0328	1.03	0.305	−0.0306	0.0979
Grade 2	0.1086	0.0421	2.58	0.010	0.0261	0.1912
Grade 3	0.0503	0.0377	1.34	0.182	−0.0235	0.1242
Grade 4	0.3289	0.0626	5.25	<0.001	0.2061	0.4516
Grade 5	0.1417	0.0425	3.34	0.001	0.0584	0.2249
Gender * sfy1	−0.0077	0.0603	−0.13	0.898	−0.1258	0.1104
Gender * thry1	−0.0329	0.1487	−0.22	0.825	−0.3243	0.2586
Gender * sfy2	0.0273	0.0388	0.71	0.481	−0.0487	0.1034
Gender * thry2	0.1144	0.0406	2.82	0.005	0.0349	0.1940
sfy1*grade 2	−0.3660	0.0995	−3.68	<0.001	−0.5611	−0.1710
sfy1*grade 3	−0.2469	0.1010	−2.44	0.015	−0.4449	−0.0489
sfy1*grade 4	−0.2428	0.1156	−2.1	0.036	−0.4694	−0.0163
sfy1*grade 5	−0.3929	0.1067	−3.68	<0.001	−0.6021	−0.1836
thry1*grade 2	0.1052	0.3160	0.33	0.739	−0.5143	0.7246
thry1*grade 3	−0.1495	0.3080	−0.49	0.627	−0.7533	0.4542
thry1*grade 4	−0.3384	0.3086	−1.1	0.273	−0.9433	0.2665
thry1*grade 5	−0.0460	0.3103	−0.15	0.882	−0.6543	0.5623
sfy2*grade 2	−0.0924	0.0529	−1.75	0.081	−0.1962	0.0113
sfy2*grade 3	0.0333	0.0487	0.68	0.495	−0.0623	0.1288
sfy2*grade 4	−0.1530	0.0708	−2.16	0.031	−0.2918	−0.0142
sfy2*grade 5	−0.0213	0.0545	−0.39	0.696	−0.1282	0.0856
thry2*grade 2	0.1085	0.0610	1.78	0.075	−0.0111	0.2281
thry2*grade 3	−0.0336	0.0502	−0.67	0.503	−0.1321	0.0648
thry2*grade 4	−0.0156	0.0670	−0.23	0.815	−0.1469	0.1157
thry2*grade 5	0.0687	0.0596	1.15	0.249	−0.0481	0.1855
Undernourishment	−0.0032	0.0013	−2.49	0.013	−0.0057	−0.0007
Net enrollment ratio	0.0052	0.0008	6.83	<0.001	0.0037	0.0067
GDP per capita	−0.0006	0.0001	−6.94	<0.001	−0.0007	−0.0004
Constant	0.5395	0.1142	4.72	<0.001	0.3157	0.7634
R-squared	0.0499					

## Discussion

This paper included the analysis of a natural experiment designed to explore the influence of school feeding programs on school enrollment, based on a meta-analysis of survey data collected in 32 countries in sub-Saharan Africa. The results indicate that school feeding programs had a positive impact on school enrollment, on the order of about 10%. As per an earlier study using this dataset (14), the changes on enrollment varied by modality of school feeding provision and by gender, with onsite meals appearing to have stronger effects in the first year of treatment in the lower primary grades, and onsite combined with take-home rations also being effective post-year one, particularly for girls that were receiving the extra take-home rations. Unlike the earlier study, this analysis provides estimates of program effect, comparing two treatment arms (onsite feeding alone and onsite feeding combined with take-home rations) to a control without intervention. These findings are consistent with those from two recent impact evaluations that also examined effects on enrollment.

As the interventions have very different program costs ($USD 50 per child per year for school meals and $USD 75 per child per year from take-home rations), these findings are potentially very relevant to policymakers looking to scale-up school feeding programs (7). However, the study findings are limited by a number of important factors and will need to be validated by further research. As highlighted in recent reviews (9), measuring impact of school feeding on enrollment would require an analysis at household and individual levels. At the school level it is not possible to control for household characteristics that are known to shape schooling decisions, such as mother’s level of education, for example (18). In addition, enrollment effects at the school level may in fact include both newly enrolled children but also transfers from other schools, making it very difficult to isolate program effects.

The operational nature of the survey data used in this meta-analysis also limits the robustness of the design and validity of the findings. The data were collected as part of WFP’s school-level monitoring of school feeding programs and not as part of an impact evaluation. Nevertheless, this analysis is the first to study possible links between enrollment and length of program duration using multivariable models, examining whether programs reach a saturation point or steady state beyond which school feeding may in fact have no further benefits on school enrollment. The estimates of program effect are consistent to those from RCT studies in the literature, suggesting that the estimation strategy provided in this paper, including school- and country-level covariates, provides a robust estimation framework. Further research is required to examine the issue of cost-effectiveness of alternative modalities in more detail.

## Conflict of Interest Statement

The author declares that the research was conducted in the absence of any commercial or financial relationships that could be construed as a potential conflict of interest.
